# Limited efficacy of a therapeutic anti-CD40 monoclonal antibody to inhibit activated CD4 T cell autoimmunity in vitro

**DOI:** 10.1371/journal.pone.0351131

**Published:** 2026-06-12

**Authors:** Chelsea Gootjes, Jaap Jan Zwaginga, Tatjana Nikolic, Bart O. Roep

**Affiliations:** Department of Internal Medicine, Section Immunomodulation and Regenerative Cell Therapy, Leiden University Medical Center, Leiden, The Netherlands; Indian Institute of Chemical Technology, INDIA

## Abstract

Iscalimab is a nondepleting anti-CD40 monoclonal antibody, expected to suppress immune responses by blocking the costimulation by antigen-presenting cells through CD40-CD40L ligation. This therapeutic antibody indeed inhibited proliferation of B lymphocytes and TNF production by dendritic cells and is being tested in clinical trials to treat B cell mediated autoimmune diseases. Since iscalimab showed limited clinical benefit and did not improve kidney and liver transplantation outcomes compared to standard tacrolimus treatment, we studied whether a biosimilar of iscalimab affects T cell responses in vitro. For this, we stimulated autoreactive effector and alloreactive naïve CD4 T cells in the presence of anti-CD40 antibody and measured the impact on cell proliferation. While we confirmed the capacity of iscalimab biosimilar antibody to bind to B cells and dendritic cells and to completely inhibit proliferating B cells, it showed limited to no efficacy to inhibit proliferation of diabetogenic beta-cell specific effector CD4 T cell clones. The CD40 blockade also did not affect the induction of proliferation of naïve alloreactive CD4 T cells, by dendritic cells. Based on our in vitro data pointing to minimal inhibition of T cells by CD40 blockade, we propose that iscalimab may not suffice to accomplish durable benefit as intervention strategy to treat type 1 diabetes or other T cell mediated diseases.

## 1. Introduction

Monoclonal antibodies are successfully used as immune therapy in different inflammatory diseases. There are more than 160 approved monoclonal antibodies used to treat tumors, organ transplantation, infectious diseases, and autoimmune diseases [1]. These include monoclonal antibodies against cytokines (e.g., anti-TNF; adalimumab, infliximab) or CD3 (e.g., teplizumab, otelixizumab), depleting monoclonal antibodies against CD20 (e.g., rituximab) and monoclonal antibodies interfering in cell-cell interactions (e.g., anti-CD25; daclizumab, anti-PD-1; nivolumab or pembrolizumab, and anti-CTLA4; ipilimumab). Additional candidate therapeutic monoclonal antibodies are under investigation for clinical use. Potential side effects can include allergic reactions, infections, platelet and thrombotic disorders, autoimmunity, dermatitis, and cardiotoxicity (reviewed in [2]).

The interaction between CD40 on B cells, monocytes and dendritic cells (DCs) and CD40L expressed on T cells is another potential target for immune intervention and will result in effector functions which are important in B and T cell activation and humoral immunity. The intracellular part of the CD40 receptor signals into antigen presenting cells (APCs) after binding to its ligand, CD40L, activating different members of adapter protein TNF receptor associated factor (TRAF) and pathways such as NFkB, MAPK, and PI3K to control cell survival, activation, and gene expression [3–5]. This is crucial for immunity, especially B cell function and DC maturation [6–8]. The CD40L signaling domain involves a short intracellular tail and transmembrane region, allowing for reverse signaling within T cell, activating pathways such as ERK and NFκB [9–11]. Since the CD40-CD40L interaction is important in multiple cellular functions, it is conceivable that blockade of this interaction could be an effective treatment to prevent transplant rejection and intervene in autoimmune diseases by preventing priming of naïve T cells. While multiple monoclonal antibodies against CD40 were developed showing prolonged allograft survival, they induced side effects like B cell depletion or partially agonistic activity [12–16].

Iscalimab (also called CFZ533) is a newly developed monoclonal antibody that binds to the cysteine-rich domain 2 (CRD2) of CD40 with antagonistic activity [17]. Iscalimab is Fc-silenced, since the Fc portion of the antibody harbors a N297A mutation preventing binding to Fc-gamma receptor and thus Fc-mediated effector functions [18], preventing cell depletion by antibody-dependent cellular cytotoxicity or complement dependent cytotoxicity, thus reducing potential side effects [19]. In vitro, iscalimab inhibited CD40L stimulated B cell proliferation and TNF production by monocyte derived DCs [19]. While these in vitro studies showed efficacy of iscalimab on B cells and DCs, effects on T cells have not been reported yet.

Iscalimab was well-tolerated and prolonged allograft survival and function in vivo in nonhuman primates [20]. However, clinical benefit was limited in clinical trials assessing iscalimab in kidney and liver transplantations (NCT03663335 and NCT03663335), and in B cell mediated autoimmune diseases such as myasthenia gravis (NCT02565576), primary Sjogren’s syndrome (NCT03905525 and NCT04541589), SLE (NCT03656562) [21–23]. Moreover, a clinical trial testing iscalimab in the T cell mediated autoimmune condition type 1 diabetes (T1D) was terminated (NCT04129528). To assess the potential efficacy or inefficacy for application in T1D, we studied whether a biosimilar of iscalimab affects antigen-specific T cell responses to islet autoantigens by inhibiting costimulation by APCs in vitro.

## 2. Materials and methods

### 2.1. Generation of monocyte-derived dendritic cells for antigen presentation

Buffy coat preparations from healthy adult peripheral blood were purchased from Sanquin (Sanquin Bloedvorziening, Amsterdam, The Netherlands), released for research under informed consent. Human peripheral blood mononuclear cells (PBMCs) were isolated from these buffy coats with Ficoll density gradient centrifugation. Monocytes were isolated to generate monocyte derived DCs (moDCs), as described in [24]. In short, monocytes were isolated using CD14 beads (130-050-201, Miltenyi) according to the manufacturer’s instructions. Isolated monocytes (2.4 x10^6 per well) were seeded in RPMI supplemented with 8% FCS, recombinant human IL-4 (500 U/mL, Miltenyi) and recombinant human GM-CSF (800 U/mL, Miltenyi) in 6-well tissue culture plates (Costar) and were incubated for 6 days at 37°C and 5% CO2. On day 3, culture medium including supplements was refreshed.

### 2.2. Anti-CD40 antibody binding to peripheral blood mononuclear cells

Iscalimab biosimilar antibody (human IgG anti-CD40, purchased from Abeomics) was used in this study (research grade antibody produced to resemble the characteristics and functionality of iscalimab). To determine the binding capacity to leukocytes, PBMCs and DCs were first pre-treated with 50% human serum, to block potential non-specific binding, after which the cells were washed with FACS buffer (PBS/ 2% fetal calf serum (FCS)) and then incubated with 10 µg/ml iscalimab for 15 minutes at room temperature. The concentration (10 µg/ml) was in line with our previous titration studies using anti-CD3 or CD25 to reach complete T-cell inhibition [25], in vitro experiments on B-cell inhibition by iscalimab innovator product by others [19], as well as trough levels in clinical trials assessing rituximab, adalimumab, tocilizumab). The unbound antibody was washed, and cells incubated with allophycocyanin (APC)- labeled mouse-anti-human IgG (5 µg/ml, clone: HP6017, Biolegend) for 15 minutes at room temperature in the dark. PBMC samples were subsequently stained with a panel of surface anti-human monoclonal antibodies consisting of: anti-CD3-APC-Cy7 (clone OKT3, Biolegend); anti-CD4-BV650 (clone SK3, BD Biosciences); anti-CD8-PE-Dazzle594 (clone SK1, Biolegend); anti-CD19-PE (clone HIB19, Biolegend); anti-CD14-AF488 (clone HCD14, Biolegend); anti-CD16-PE-Cy7 (clone 3G8, Biolegend); anti-CD15-BV605 (clone W6D3, Biolegend), and anti-CD56-BV510 (clone 16.2, BD Biosciences) to identify different leukocyte subsets. Samples were acquired on an Aurora 3 laser spectral flow cytometer (Cytek) or Canto (BD Bioscience) and analyzed using FlowJo (version 10.9.0). Dead cells were excluded from the analysis. T cells were gated as SSClow and CD3positive, B cells as SSClow and CD19positive, and monocytes as CD14 positive and CD16positive.

### 2.3. B cell inhibition

Human B cells were isolated from buffy coat PBMCs by negative selection using the Miltenyi isolation kit Pan B cell isolation kit (130-101-638) according to the manufacturer’s instructions. B cells (1 x 10^5^/well) were stimulated with IL-4 (75 ng/ml, Miltenyi) and CD40L (5 µg/ml, Miltenyi) only or in the presence of iscalimab (two-fold dilutions ranging between 10–0.019 µg/ml) in IMDM medium supplemented with 8%FCS. B cells stimulated with IL-4/CD40L without iscalimab were taken along as positive control and without IL-4/CD40L stimulation as negative control. B cells were incubated for 4 days at 37°C and 5% CO2. The final 6 hours of incubation 3H-thymidine (10 µCi/ml) was added, after B cells were harvested (Tomtec harvester 96 Mach II) and radioactivity was measured as counts per minute (CPM) using a MicroBeta2 2450 Microplate counter (PerkinElmer, Waltham, US). CPM values were normalized according to the positive (100) and negative (0) controls.

### 2.4. Inhibition of proliferation of autoreactive effector CD4 T cell clones

The beta-cell antigen specific CD4 + T clones used in this study were described previously [26–31], including an HLA-DR3 restricted CD4 clone against GAD65 (PM1#11; GAD65-epitope TVYGAFDPLLAVAD), a HLA-DR1 restricted T-cell clone responding to the Imogen 38 protein (mitochondrial islet autoantigen; 1C6; epitope LWEIEFAKQL), a T-cell clone against the insulin B-chain (Clone 5; epitope LCGSHLVEALYLVCGER) in HLA-DQ8, and a T cell clone against proinsulin, recognizing an epitope spanning the A- and C-chain of proinsulin (2.13; epitope C19-A3, GSLQPLALEGSLQKRGIV) in HLA-DR4. This panel of islet autoreactive CD4 clonal T cells was stimulated with irradiated (3500 rad) PBMCs expressing the appropriate HLA class II restriction molecules (ratio 1:5) in IMDM medium supplemented with 10% human serum (HS), alone or with a peptide dilution series appropriate for each clone, in triplicate. For each condition, and additional triplicate was treated with anti-CD40 antibody (10 µg/ml). Cells were co-cultured for 3 days at 37°C and 5% CO2, after which 3H-thymidine (10 µCi/ml) was added to the co-culture for 16–18 hours. Cells were harvested and radioactivity was measured as described in our B cell inhibition experiment. CD4 T cells clones were stained for CD40L in resting state (after thawing) or after stimulation with anti-CD3 (UCHT1, 10 µg/ml) for 3 days. Clones were stained with anti-CD40L- PE-CF594 (clone TRAP1, BD Bioscience) for 15 minutes at room temperature, washed and analyzed on an Aurora 3 laser spectral flow cytometer (Cytek). Data was analyzed using FlowJo (version 10.9.0).

### 2.5. Allogeneic T-cell reactivity by mixed lymphocyte reaction (MLR)

Immature dendritic cells of two donors were matured with GM-CSF (800 IU/ml, Miltenyi), IL-6 (500 IU/ml, Miltenyi), IL-1beta (1600 IU/ml, Miltenyi), TNF-alpha (335 IU/ml, Miltenyi), and PGE2 (40 µg/ml, Miltenyi) in RPMI medium supplemented with 8% FCS for 2 days. Mature DCs were then harvested and pooled together and used as stimulator of the HLA mismatched T cells. DCs were co-cultured in IMDM medium supplemented with 10% HS, with either peripheral blood lymphocytes (PBL, ratio 1:20) or isolated naïve CD4 T cells (ratio 1:5), from six different donors in triplicate. Naïve CD4 T cells were isolated with MACS naïve CD4 T cell isolation kit II (130-094-131) according to the manufacturer’s instructions. Cells were co-cultured alone or in the presence of anti-CD40 antibody (10 µg/ml) for 4 days at 37°C and 5% CO2. To control for inhibition of the MLR response, we used an HLA class II blocking antibody (10 µg/ml; clone Tü39, Biolegend). 3H-thymidine (10 µCi/ml) was added for 16–18 hours before harvest and radioactivity was measured as described above.

### 2.6. Data analysis

Flow cytometry data was analyzed using FlowJo (version 10.9.0). Statistical significance was tested using GraphPad Prism (version 8.0.2). T cell clones stimulated with (red line) or without (blue line) iscalimab biosimilar were compared, where proliferation rates (raw CPM values) were log-transformed and tested in a two-way ANOVA corrected for multiple comparisons with a Sidak test. Paired student T test (one-sided, expecting reduction) was used to test for inhibition of T cell activation in the presence of iscalimab biosimilar. P value < 0.05 was considered significant.

## 3. Results

### 3.1. Anti-CD40 antibody binds to dendritic cells, monocytes and B cells but not to T cells

**Fig 1:**
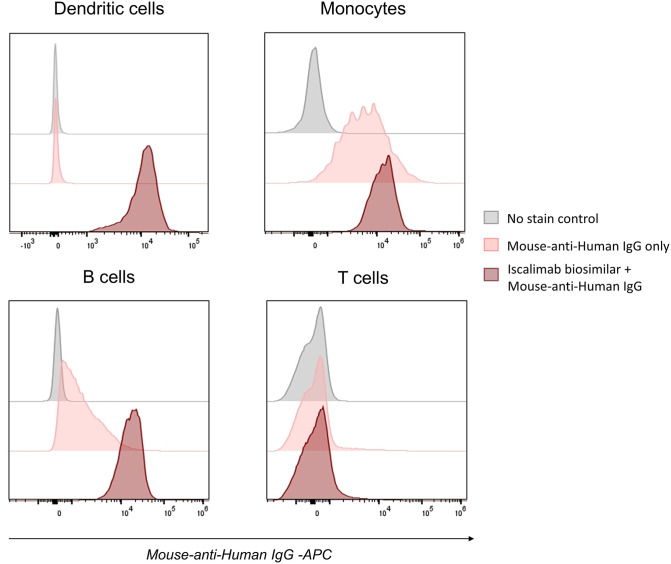
Iscalimab biosimilar binds to dendritic cells, monocytes, and B cells but not to T cells. **Cells were incubated with iscalimab biosimilar for 15 min at room temperature, followed by fluorescently APC-labeled secondary antibody.** No stain control (grey), Mouse-anti-Human IgG APC only (pink) and Iscalimab biosimilar followed by Mouse-anti-Human IgG APC (red).

First, we analyzed whether the iscalimab biosimilar binds to T cells, using flow cytometry analysis. Dendritic cells, monocytes and B cells showed a strong and specific binding of the monoclonal antibody whereas, as expected, no binding was observed when incubated with T cells (Fig 1).

### 3.2. Anti-CD40 antibody inhibits B cell proliferation in a dose-dependent manner

**Fig 2:**
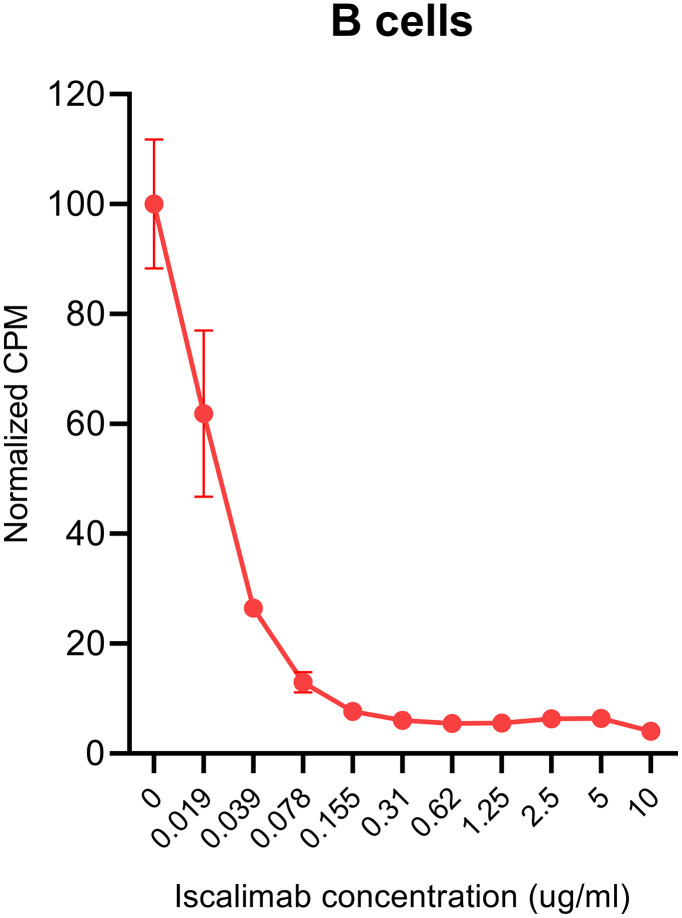
Iscalimab biosimilar can inhibit the proliferation of B cells in a dose-dependent manner. **B cells were stimulated with CD40L and IL-4 in the presence of a range of Iscalimab concentrations.** B cells stimulated with IL-4/CD40L without Iscalimab were taken along as positive control and without IL-4/CD40L stimulation as negative control. CPM values were normalized according to the positive (100) and negative (0) controls. Symbols represent an average of a triplicate and the standard deviation.

Previously, studies reported that iscalimab inhibits B cells in vitro. Hence, we stimulated B cells with CD40L and IL-4 alone or in the presence of a range of iscalimab concentrations and analyzed the proliferation after 4 days. Indeed, more than 90% inhibition of the B cell proliferation was observed at a concentration of 0.1 µg/ml, confirming that this iscalimab biosimilar antibody inhibits B cell proliferation effectively, as reported previously (Fig 2).

### 3.3. Anti-CD40 antibody does not inhibit proliferation of autoreactive effector CD4 T cell clones

To test whether the iscalimab-biosimilar is able to inhibit pre-existing autoreactive T cell responses by interfering in costimulation by DCs, we tested a panel of four beta-cell specific effector CD4 T cell clones with different HLA restrictions, recognizing different islet autoantigens and epitopes and with different proliferative capacity in response to autoantigen-specific stimulation. To maximize the effect, we chose to use an iscalimab concentration (10 µg/ml), which was hundred times higher than the concentration sufficient to abolish B cells proliferation and was in line with our previous titration studies using anti-CD3 or -CD25 to reach complete T-cell inhibition [25], as well as trough levels in clinical trials assessing rituximab, adalimumab, tocilizumab.

**Fig 3:**
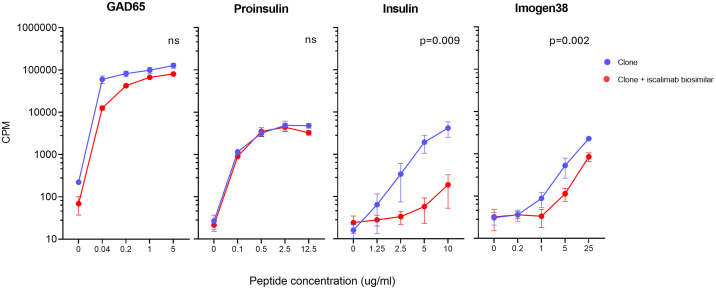
Anti-CD40 antibody does not prevent proliferation of autoreactive effector CD4 T cell clones. CD4 clones against GAD65 (PM1#11; HLA-DR3 restricted), proinsulin (2.13; HLA-DR4 restricted), insulin B-chain (Clone 5; HLA-DQ8 restricted), and Imogen 38 protein (mitochondrial islet autoantigen; 1C6, HLA-DR1 restricted) were stimulated with irradiated PBMCs (expressing the appropriate HLA molecules) in the presence of a peptide dilution series appropriate for each clone, alone (blue line), or with the addition of iscalimab biosimilar (10 µg/ml, red line). Symbols represent an average of a triplicate and the standard deviation. Clones stimulated with (red line) or without (blue line) the presence of iscalimab biosimilar were compared, where proliferation rates (raw CPM values) were log-transformed and tested in a two-way ANOVA corrected for multiple comparisons with a Sidak test.

The proliferation of two high-affinity T cells clones was unaffected (PM1#11: p = 0.23 and clone 2.13: p = 0.19), while there was a limited decrease in proliferation in the presence of iscalimab for lower-affinity T cell clones (InsB6-22: p = 0.009 and 1C6: p = 0.002) that require higher peptide concentrations for a proliferative response (Fig 3). This limited decrease in proliferation for T cell clones with lower-affinity was independent of their CD40L expression, as the abundance of clones expressing CD40L varied in resting state (14.5%, 15.6%, 6.1% and 39.9% for clones PM1#11, 2.13, InsB6-22 and 1C6, respectively). CD40L expression increased after anti-CD3 stimulation (94.9%, 71.7% and 89.6%, for clones PM1#11, 2.13 and 1C6, respectively), indicating that CD40L expression increased in T cell clones after stimulation, which was independent of the rate of their inhibition by iscalimab biosimilar.

### 3.4. Anti-CD40 antibody failed to inhibit proliferation of alloreactive naïve CD4 T cells

**Fig 4:**
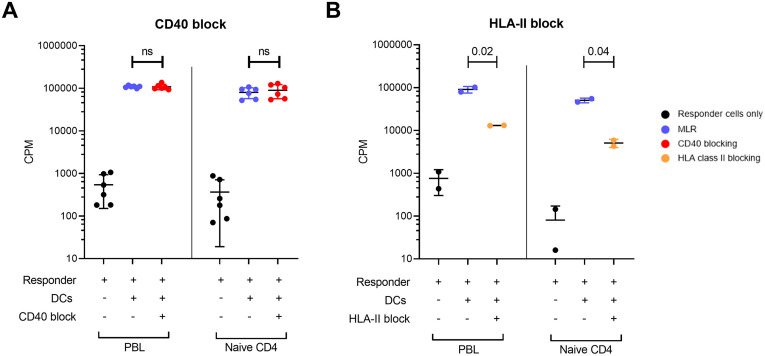
Anti-CD40 antibody (10 µg/ml) does not influence the activation of alloreactive naïve CD4 T cells. Responder cells, (either peripheral blood lymphocytes (PBL) or naïve CD4 T cells) were cultured with a mix of mature DCs (2 donors) in a mixed lymphocyte reaction assay (MLR), with CD40 blocking antibody (A) or HLA class II blocking antibody **(B)**. Responder cells only (black), MLR (blue), CD40 block (red) and HLA class II block (orange). Symbols represent different donors and mean with standard deviation are shown. Paired student T test (one sided, assessing changes in one direction only) was used to test for inhibition of T cell activation in the presence of iscalimab biosimilar or HLA class II blocking.

To test whether iscalimab inhibits the priming of naïve CD4 T cells responses, we cultured mature monocyte-derived dendritic cells with total peripheral blood lymphocytes or isolated naïve CD4 T cells in a mixed lymphocyte reaction assay (MLR; Fig 4). Both total lymphocytes (PBL) and isolated naïve CD4 T cells elicited a strong proliferative response to the allogeneic dendritic cells, which remained unaffected in the presence of the iscalimab biosimilar (Fig 4A). In contrast, a similar concentration of monoclonal antibodies against HLA class II effectively blocked the MLR response of PBL (85%) and isolated naïve CD4 T cells (90%; Fig 4B).

## 4. Discussion

Iscalimab, a CD40 antagonist, has been tested in clinical trials as a treatment for B cell and autoantibody mediated autoimmune diseases such as myasthenia gravis, primary Sjogren’s syndrome and SLE with limited clinical benefit, while a clinical trial in T1D was stopped [21–23]. In vitro studies have shown an effect of iscalimab on B cell proliferation and cytokine production by DCs, but antagonistic effects for T cell responses have not been reported so far. We confirm that an iscalimab biosimilar antibody bound to CD40 expressing cells (DCs, monocytes and B cells). While it has been reported that some activated CD4 and CD8 T cells can express CD40 [32], no binding of the iscalimab biosimilar to T cells was observed in our study, indicating that effects of iscalimab on T cells are likely induced through antigen-presenting cells.

We further confirmed the ability of iscalimab biosimilar to strongly inhibit B cell proliferation at low antibody concentrations. Yet, T cell proliferation was hardly affected in vitro. Two high-affinity T cell clones were completely unaffected by anti-CD40 blockade, while two other autoreactive T cell clones requiring high epitope concentrations for the response (i.e., lower-affinity), reduced T cell proliferation only mildly in the presence of iscalimab. Of note, the iscalimab concentration used in these experiments was hundred times higher than de concentration needed to abolish B cell proliferation. This reduction was independent of the rate of CD40L expression by CD4 T cells clone cells.

The absence of an inhibitory effect may not be surprising in the case of primed effector CD4 T cell clones, which are less dependent on costimulation. We anticipated that the impairing effect of iscalimab on T cells would be particularly notable on priming of naïve CD4 T cell responses since this process critically requires costimulation provided by antigen presenting cells [33,34]. It is conceivable that preventing priming of T cell autoimmunity at disease onset could be too late if preactivated T cell autoimmunity would be unaffected. Yet, blocking another costimulatory molecule (CD80/CD86 with abatacept) in recent-onset type 1 diabetes patients prolonged beta cell function compared to the placebo group [35]. This may result from preventing new naïve T cells that could contribute to so-called epitope spreading to become activated. Indeed, we demonstrated that longer disease duration in type 1 diabetes was associated with T cell responses to additional islet antigens [36], which may be prevented by costimulation blockade even long after initiation of the disease process or diagnosis. This may also apply to iscalimab. Nonetheless, we here demonstrate that naïve CD4 allogeneic T cells were unaffected even by the highest tested concentration of CD40 blocking antibody, whereas HLA class II blockade did inhibit alloreactive naïve CD4 T cells. Given that CD40 stimulation is important for CD8 T cell priming [37,38], blocking CD40 may still interfere in CD4 T cell help to CD8 T cells, thus preventing CD8 T cell activation.

Iscalimab has already been tested in clinical trials in B cell mediated autoimmune diseases. In myasthenia gravis, iscalimab was assessed as an add-on therapy in patients with moderate-to-severe myasthenia gravis [21]. So far, differences in disease scores (the primary endpoint) between placebo and treated patients were not achieved, implying that iscalimab is not a suitable add-on therapy in myasthenia gravis. Yet, in Sjogren’s syndrome, the first placebo-controlled proof-of-concept study showed that intravenous treatment with iscalimab (10 mg/kg) reduced the disease score compared to placebo, although subcutaneous injection (3 mg/kg) did not show a difference [23]. However, the sample size was small (8 patients treated subcutaneous and 20 intravenous) and the trial of short duration (24 weeks). Another phase 2b dose-finding study of small sample size in Sjogren’s syndrome concluded that there was preliminary effectivity of iscalimab after 24 weeks in two distinct patient populations [22]. A clinical trial in SLE is still ongoing. Overall, clinical data in support of use of iscalimab in autoimmune diseases is meager and a clinical benefit in B cell mediated autoimmune diseases remains to be demonstrated.

In clinical transplantation, iscalimab showed no added clinical benefit. While iscalimab showed prolonged allograft survival and function in nonhuman primates [20], an interim analysis of a clinical trial in kidney transplantation (NCT03663335) revealed that iscalimab was less efficacious in kidney graft survival as compared to the standard tacrolimus treatment, which led to discontinuation of this trial. Iscalimab was also tested in liver transplantations (NCT03781414), but this trial was halted since no effect of iscalimab in kidney transplantation was observed.

The clinical efficacy of iscalimab may be impacted by dosing, pharmacokinetics, patient and disease heterogeneity, disease stage, or affected tissue. Our data add an alternative explanation why iscalimab showed limited clinical benefit in T cell mediated diseases, where CD40 blockade would be insufficient to offer durable benefit as intervention strategy, as has been shown in clinical trials in transplantation and T1D. Yet, this notion would not rule out that alternative strategies interrupting CD40-CD40L interactions could prove successful. Blocking CD40L in mice induced CD4 T cell tolerance and linked suppression [39] and prevented CD8 priming [37], and thus also interfere in epitope spreading in autoimmune diseases and graft rejection. Indeed, an antagonistic CD40L monoclonal antibody (frexalimab) is currently being tested to preserve beta-cell function in newly diagnosed T1D patients (NCT06111586), whereas another anti-CD40L antagonist was withdrawn in several clinical trials for different indication due to uncertainties regarding its safety in humans, while the antibody showed potential in preclinical models.

## Supporting information

S1 File Flow Cytometry DCs. (ZIP)

S2 File Flow Cytometry PBMCs Donor 1. (ZIP)

S3 File Flow Cytometry PBMCs Donor 2. (ZIP)

S4 File Flow Cytometry PBMCs Donor 3. (ZIP)

S5 File Flow Cytometry PBMCs Donor 4. (ZIP)

S6 File Flow Cytometry PBMCs Donor 5. (ZIP)

S7 File Flow Cytometry PBMCs Donor 6. (ZIP)
