# Supplementary material for: Limited efficacy of a therapeutic anti-CD40 monoclonal antibody to inhibit activated CD4 T cell autoimmunity in vitro
Source: PLoS One. 2026 Jun 12;21(6):e0351131. doi: 10.1371/journal.pone.0351131 (PMC13262827; doi:10.1371/journal.pone.0351131)
Supplement: Peer review history

# Peer review: Decision Letter 0

**DOI:** 10.1371/journal.pone.0351131.r001

**Authors:** Srinivasa Reddy Bonam

20 Jan 2026

PONE-D-25-64177

Limited efficacy of a therapeutic anti-CD40 monoclonal antibody to inhibit activated CD4 T cell autoimmunity in vitro

PLOS One

Dear Dr. Bart O. Roep,

Thank you for submitting your manuscript to PLOS ONE. We have requested three worthy reviewers to comment on your study, and they feel that it has merit but does not fully meet PLOS ONE’s publication criteria as it currently stands. Therefore, we invite you to consider the reviwers comments and submit a revised version of the manuscript that addresses the points raised during the review process.

Please submit your revised manuscript by Mar 05 2026 11:59PM. If you will need more time than this to complete your revisions, please reply to this message or contact the journal office at plosone@plos.org. When you're ready to submit your revision, log on to https://www.editorialmanager.com/pone/ and select the 'Submissions Needing Revision' folder to locate your manuscript file.

Please include the following items when submitting your revised manuscript:

- A letter that responds to each point raised by the academic editor and reviewer(s). You should upload this letter as a separate file labeled 'Response to Reviewers'.
- A marked-up copy of your manuscript that highlights changes made to the original version. You should upload this as a separate file labeled 'Revised Manuscript with Track Changes'.
- An unmarked version of your revised paper without tracked changes. You should upload this as a separate file labeled 'Manuscript'.

If you would like to make changes to your financial disclosure, please include your updated statement in your cover letter. Guidelines for resubmitting your figure files are available below the reviewer comments at the end of this letter.

-->If applicable, we recommend that you deposit your laboratory protocols in protocols.io to enhance the reproducibility of your results. Protocols.io assigns your protocol its own identifier (DOI) so that it can be cited independently in the future. For instructions see: https://journals.plos.org/plosone/s/submission-guidelines#loc-laboratory-protocols. Additionally, PLOS ONE offers an option for publishing peer-reviewed Lab Protocol articles, which describe protocols hosted on protocols.io. Read more information on sharing protocols at https://plos.org/protocols?utm_medium=editorial-email&utm_source=authorletters&utm_campaign=protocols.

We look forward to receiving your revised manuscript.

Kind regards,

Srinivasa Reddy Bonam

Academic Editor

PLOS One

Journal Requirements:

When submitting your revision, we need you to address these additional requirements.

1. Please ensure that your manuscript meets PLOS ONE's style requirements, including those for file naming. The PLOS ONE style templates can be found at

https://journals.plos.org/plosone/s/file?id=wjVg/PLOSOne_formatting_sample_main_body.pdf and

https://journals.plos.org/plosone/s/file?id=ba62/PLOSOne_formatting_sample_title_authors_affiliations.pdf

2. Thank you for stating in your Funding Statement:

“The authors are supported by Stichting DON.”

Please provide an amended statement that declares *all* the funding or sources of support (whether external or internal to your organization) received during this study, as detailed online in our guide for authors at http://journals.plos.org/plosone/s/submit-now. Please also include the statement “There was no additional external funding received for this study.” in your updated Funding Statement.

Please include your amended Funding Statement within your cover letter. We will change the online submission form on your behalf.

3. Thank you for stating the following financial disclosure:

“The authors are supported by Stichting DON.”

Please state what role the funders took in the study. If the funders had no role, please state: "The funders had no role in study design, data collection and analysis, decision to publish, or preparation of the manuscript."

If this statement is not correct you must amend it as needed.

Please include this amended Role of Funder statement in your cover letter; we will change the online submission form on your behalf.

4. In the online submission form, you indicated that the raw data supporting the conclusions of this article will be made available by the authors on request.

All PLOS journals now require all data underlying the findings described in their manuscript to be freely available to other researchers, either 1. In a public repository, 2. Within the manuscript itself, or 3. Uploaded as supplementary information.

This policy applies to all data except where public deposition would breach compliance with the protocol approved by your research ethics board. If your data cannot be made publicly available for ethical or legal reasons (e.g., public availability would compromise patient privacy), please explain your reasons on resubmission and your exemption request will be escalated for approval.

5. If the reviewer comments include a recommendation to cite specific previously published works, please review and evaluate these publications to determine whether they are relevant and should be cited. There is no requirement to cite these works unless the editor has indicated otherwise.

6. Please review your reference list to ensure that it is complete and correct. If you have cited papers that have been retracted, please include the rationale for doing so in the manuscript text, or remove these references and replace them with relevant current references. Any changes to the reference list should be mentioned in the rebuttal letter that accompanies your revised manuscript. If you need to cite a retracted article, indicate the article’s retracted status in the References list and also include a citation and full reference for the retraction notice.

Reviewers' comments:

Reviewer's Responses to Questions

**Comments to the Author**

1. Is the manuscript technically sound, and do the data support the conclusions?

The manuscript must describe a technically sound piece of scientific research with data that supports the conclusions. Experiments must have been conducted rigorously, with appropriate controls, replication, and sample sizes. The conclusions must be drawn appropriately based on the data presented.

Reviewer #1: Yes

Reviewer #2: Partly

Reviewer #3: Yes

**********

2. Has the statistical analysis been performed appropriately and rigorously?

Reviewer #1: No

Reviewer #2: Yes

Reviewer #3: No

**********

3. Have the authors made all data underlying the findings in their manuscript fully available?

The PLOS Data policy requires authors to make all data underlying the findings described in their manuscript fully available without restriction, with rare exception (please refer to the Data Availability Statement in the manuscript PDF file). The data should be provided as part of the manuscript or its supporting information, or deposited to a public repository. For example, in addition to summary statistics, the data points behind means, medians and variance measures should be available. If there are restrictions on publicly sharing data—e.g. participant privacy or use of data from a third party—those must be specified.

Reviewer #1: Yes

Reviewer #2: Yes

Reviewer #3: No

**********

4. Is the manuscript presented in an intelligible fashion and written in standard English?

PLOS ONE does not copyedit accepted manuscripts, so the language in submitted articles must be clear, correct, and unambiguous. Any typographical or grammatical errors should be corrected at revision, so please note any specific errors here.

Reviewer #1: Yes

Reviewer #2: Yes

Reviewer #3: Yes

**********

5. Review Comments to the Author

Please use the space provided to explain your answers to the questions above. You may also include additional comments for the author, including concerns about dual publication, research ethics, or publication ethics. (Please upload your review as an attachment if it exceeds 20,000 characters)

Reviewer #1: Introduction with adequate flow, starting with how so many monoclonal antibodies are approved therapies, then zooming into the CD40-CD40L interaction and then finally Iscalimab, a non-depleting anti-CD40 antibody. Previous work had shown that Iscalimab blocks B cell proliferation and TNF production by dendritic cells as expected. Here the authors focus instead on T cells, evaluating the impact of a Iscalimab biosimilar in naïve T cell co-cultures with donor mismatched dendritic cells. However, it is missing a very important piece: mentioning that, just like CD40, CD40L has signaling activity. Clarifying that in the introduction will provide the rationale of the whole study. CD40L on T cells binding to CD40 on B cells and giving them help is very well know, but reverse signaling on T cells by engaging CD40L is not. Please provide a short description of signaling via CD40 and via CD40L and accompanying references in the Introduction. The intracellular part of the CD40 receptor relays signals into the cell after its ligand CD40L binds, activating pathways like NFkB, MAPK, and PI3K to control cell survival, activation, and gene expression, crucial for immunity, especially B-cell function and DC maturation, using TRAF proteins to link to downstream kinases and transcription factors. The CD40L signaling domain involves its short intracellular tail and transmembrane region, allowing for reverse signaling within the T cell itself, activating pathways like ERK and NFκB.

“it is conceivable that that blockade” – repetition of “that” (typo)

Figure 1 – proper controls were used to distinguish between specific binding and non-specific binding. This was important as monocytes and B cells have significant background, whereas dendritic cells and T cells do not.

“as expected, no binding was observed when incubated with T cells”

-The authors should include PMID: 12242444 in the Discussion as a reference that Activated CD4+and CD8+ T cells express CD40

For Figure 3 and Figure 4, the authors should consider including a legend for the colors in the figure itself in addition to the text.

More importantly, Figure 1, Figure 3, and Figure 4 are missing statistics. Figure 1 could compare media fluorescence intensity from replicates to show whether levels of CD40 are statistically significantly different between control and biosimilar staining. Figure 2 statistical significance between red line and blue line, and for Figure 4 statistical significance in difference between blue and red (left) and between blue and yellow (right). Without statistics one cannot claim presence of absence of differences.

In Figure 3 it appears that there’s a difference between red and blue line for the two clones on the right, but not for the two clones on the left. The authors should provide CD40L expression data for the 4 clones in Figure 3, as well as for the T cells used in Figure 4. A simple explanation for the lack of effect of CD40 antagonist would be that the T cells don’t express or express low levels of CD40L and hence would be unaffected from blocking CD40 on antigen presenting cells.

Reviewer #2: 1. Authors need to add justification on why iscalimab biosimilar is being used and not iscalimab innovator product (Code: CFZ533) which is not yet approved by the regulatory authorities.

2. The authors must also explicitly state that iscalimab biosimilar that is used for the study has been characterized with the iscalimab innovator product (Code: CFZ533) in terms of structure and function.

3. The authors must also disclose if the studies mentioned in the article (lines 61 to 65) were performed with iscalimab biosimilar or with innovator product.

4. The authors can discuss in depth the impact of this study on the available clinical trial finding in discussion section as additional paragraph. Since, this study justifies why Iscalimab failed in clinical trials, this justification will help the reader.

5. The authors mention funding from Stichting DON but does not disclose whether Iscalimab biosimilar was gifted or bought from the Abeomics.

6. The authors mention code CFZ-533 for Iscalimab biosimilar which is also given for Novartis Innovator product. The authors need to rectify this statement.

Reviewer #3: The manuscript examines whether a biosimilar of anti‑CD40 (iscalimab) modulates effector and naive CD4+ T-cell proliferation in vitro, confirming B-cell inhibition but finding no effect on T-cell responses. The topic is timely and relevant. However, limitations in design, validation, and reporting make it difficult to draw strong mechanistic conclusions. A substantive revision is required before the work can be considered for publication.

Abstract:

Starting with the abstract, which draws a strong clinical conclusion i.e, “iscalimab is unlikely to offer durable benefit as intervention strategy to treat T cell mediated diseases” based solely on in vitro data. This overinterprets the findings. The conclusions should be toned down and framed as hypothesis-generating, with explicit acknowledgment of the limitations of in vitro systems and the heterogeneous outcomes observed in clinical studies.

Introduction:

The narrative currently relies on clinical trial failures to justify the study and could benefit from a more thorough discussion of alternative explanations (e.g., dosing, pharmacokinetics, patient heterogeneity, disease stage, or tissue targeting). The premise that lack of T-cell inhibition is the main reason for clinical failure is somewhat speculative. Broadening the discussion of CD40 complexity and explicitly acknowledging that clinical inefficacy may result from multiple factors beyond T-cell costimulation would provide valuable context.

Methods:

Antibody concentration: For PBMC and DC staining, 10 µg/mL iscalimab was used, which is a very high concentration. Please justify this choice. Was a titration performed to determine the minimal concentration required for effective staining?

Viability: Did the authors assess cell viability and exclude dead cells during flow cytometry analysis, as it is essential to avoid artifactual results?

Isotype control: Was a proper isotype control antibody of the same class and at the same concentration used to define positive gates and exclude nonspecific binding? Including such controls is crucial for validating specificity.

Secondary antibody: Report the concentration of Mouse‑anti‑Human IgG APC secondary antibody used for staining.

T Cell Models: The study used four long-term cultured, β-cell-specific, autoreactive CD4 T-cell clones. While these are well defined, they represent highly selected, homogeneous effector populations that may not fully reflect the diversity, differentiation states, and costimulatory requirements of T cells in vivo. This selection could influence the detection of CD40 blockade effects and thus the generalisability of the conclusions. Including more physiologically relevant models (e.g., polyclonal or freshly isolated patient T cells) or explicitly acknowledging that this model may underestimate the potential effects of CD40 blockade on other T-cell subsets could further strengthen the study's impact.

B Cell Inhibition: If possible, include comparative controls (e.g., other CD40‑blocking agents) to contextualise potency and specificity.

Statistics: One-sided paired t-test is used without strong justification, which is generally discouraged unless a clear, a priori directional hypothesis is prespecified. The authors should consider using two-sided tests and apply multiple-comparison adjustments where applicable.

Results and Figures:

Figures 1, 3, and 4 lack colour legend entries and should be corrected.

In Figure 1 (monocyte panel), the secondary antibody (mouse-anti-human IgG-APC) shows substantial non-specific binding relative to the no stain control. This should be addressed. Include representative flow plots showing isotype control-based gating and provide the full gating strategy as a supplementary figure.

In Section 3.2 (line 170): The claim of “almost complete inhibition at 0.1 µg/mL” overstates the data in Figure 2 and should be revised for accuracy.

Discussion:

Some aspects of the discussion may benefit from a more balanced interpretation. Conclusions appear to extend beyond what the in vitro data support and may attribute clinical failures primarily to T-cell insensitivity, which remains speculative. The authors should emphasise that the findings are hypothesis generating, clearly distinguish observations from clinical interpretation, and thoughtfully discuss the possible roles of in vivo factors (such as inflammation, tissue niches, and timing) in modulating CD40 dependence and therapeutic outcomes.

**********

6. PLOS authors have the option to publish the peer review history of their article (what does this mean?). If published, this will include your full peer review and any attached files.

If you choose “no”, your identity will remain anonymous but your review may still be made public.

**Do you want your identity to be public for this peer review?** For information about this choice, including consent withdrawal, please see our Privacy Policy.

Reviewer #1: No

Reviewer #2: No

Reviewer #3: **Yes:** Md Jahangir Alam

**********

[NOTE: If reviewer comments were submitted as an attachment file, they will be attached to this email and accessible via the submission site. Please log into your account, locate the manuscript record, and check for the action link "View Attachments". If this link does not appear, there are no attachment files.]

To ensure your figures meet our technical requirements, please review our figure guidelines: https://journals.plos.org/plosone/s/figures

You may also use PLOS’s free figure tool, NAAS, to help you prepare publication quality figures: https://journals.plos.org/plosone/s/figures#loc-tools-for-figure-preparation.

NAAS will assess whether your figures meet our technical requirements by comparing each figure against our figure specifications.

# Author comment: Author response to Decision Letter 1

**DOI:** 10.1371/journal.pone.0351131.r002

20 Apr 2026

Reviewer #1

Introduction with adequate flow, starting with how so many monoclonal antibodies are approved therapies, then zooming into the CD40-CD40L interaction and then finally Iscalimab, a non-depleting anti-CD40 antibody. Previous work had shown that Iscalimab blocks B cell proliferation and TNF production by dendritic cells as expected. Here the authors focus instead on T cells, evaluating the impact of a Iscalimab biosimilar in naïve T cell co-cultures with donor mismatched dendritic cells. However, it is missing a very important piece: mentioning that, just like CD40, CD40L has signaling activity. Clarifying that in the introduction will provide the rationale of the whole study. CD40L on T cells binding to CD40 on B cells and giving them help is very well know, but reverse signaling on T cells by engaging CD40L is not. Please provide a short description of signaling via CD40 and via CD40L and accompanying references in the Introduction. The intracellular part of the CD40 receptor relays signals into the cell after its ligand CD40L binds, activating pathways like NFkB, MAPK, and PI3K to control cell survival, activation, and gene expression, crucial for immunity, especially B-cell function and DC maturation, using TRAF proteins to link to downstream kinases and transcription factors. The CD40L signaling domain involves its short intracellular tail and transmembrane region, allowing for reverse signaling within the T cell itself, activating pathways like ERK and NFκB.

>> We thank the reviewer for this suggestion allowing us to improve our introduction. A short description of signaling via CD40 and CD40L is added to the introduction (page 2).

“The intracellular part of the CD40 receptor signals into antigen presenting cells (APCs) after binding to its ligand, CD40L, activating different members of adapter protein TNF receptor associated factor (TRAF) and pathways such as NFkB, MAPK, and PI3K to control cell survival, activation, and gene expression [3-5]. This is crucial for immunity, especially B cell function and DC maturation [6-8]. The CD40L signaling domain involves a short intracellular tail and transmembrane region, allowing for reverse signaling within T cell, activating pathways such as ERK and NFκB [9-11].”

“it is conceivable that that blockade” – repetition of “that” (typo)

>> Thank you; we corrected this typo.

Figure 1 – proper controls were used to distinguish between specific binding and non-specific binding. This was important as monocytes and B cells have significant background, whereas dendritic cells and T cells do not.

“as expected, no binding was observed when incubated with T cells” -The authors should include PMID: 12242444 in the Discussion as a reference that Activated CD4+and CD8+ T cells express CD40

>>Indeed activated CD4 and CD8 T cells can express CD40. This observation has been added to the discussion (page 10).

“We confirm that an iscalimab biosimilar antibody bound to CD40 expressing cells (DCs, monocytes and B cells). While it has been reported that some activated CD4 and CD8 T cells can express CD40 [32], no binding of the iscalimab biosimilar to T cells was observed in our study, indicating that effects of iscalimab on T cells are likely induced through antigen-presenting cells.”

For Figure 3 and Figure 4, the authors should consider including a legend for the colors in the figure itself in addition to the text.

>> Thank you for this suggestion. We have now included color legends in the figures themselves.

More importantly, Figure 1, Figure 3, and Figure 4 are missing statistics. Figure 1 could compare media fluorescence intensity from replicates to show whether levels of CD40 are statistically significantly different between control and biosimilar staining. Figure 2 statistical significance between red line and blue line, and for Figure 4 statistical significance in difference between blue and red (left) and between blue and yellow (right). Without statistics one cannot claim presence of absence of differences.

>> Thank you for raising this point. We now performed statistical testing for Figures 3 and 4, which is reported in the revised manuscript (page 6). For Figure 1, statistics would seem less relevant, since we intended to report qualitative differences rather than quantitative, between controls (secondary antibody only) and the biosimilar plus secondary antibody; the histograms provide compelling support that indeed iscalimab binds B cells.

“Flow cytometry data was analyzed using FlowJo (version 10.9.0). Statistical significance was tested using GraphPad Prism (version 8.0.2). T cell clones stimulated with (red line) or without (blue line) iscalimab biosimilar were compared, where proliferation rates (raw CPM values) were log-transformed and tested in a two-way ANOVA corrected for multiple comparisons with a Sidak test. Paired student T test (one -sided, expecting reduction) was used to test for inhibition of T cell activation in the presence of iscalimab biosimilar. P value < 0.05 was considered significant.”

In Figure 3 it appears that there’s a difference between red and blue line for the two clones on the right, but not for the two clones on the left. The authors should provide CD40L expression data for the 4 clones in Figure 3, as well as for the T cells used in Figure 4. A simple explanation for the lack of effect of CD40 antagonist would be that the T cells don’t express or express low levels of CD40L and hence would be unaffected from blocking CD40 on antigen presenting cells.

>> We appreciate that providing CD40L expression on our clones would help explaining the lack of effect of CD40 antagonist. We measured CD40L expression on our clones in resting state and after activation (anti-CD3 stimulation). Indeed, the abundance of CD40L expressing cells between T cell clones varied in resting state (6-39%). Yet, after anti-CD3 stimulation the abundance increased in all clones (72-95%), indicating that all clones could be inhibited by iscalimab biosimilar, and implying that any T cell inhibition was unrelated to the rate of their CD40L expression. CD40L expression by our T clones is now reported in the revised manuscript (page 8).

Reviewer #2

1. Authors need to add justification on why iscalimab biosimilar is being used and not iscalimab innovator product (Code: CFZ533) which is not yet approved by the regulatory authorities.

>>The iscalimab innovator product was not available to us.

2. The authors must also explicitly state that iscalimab biosimilar that is used for the study has been characterized with the iscalimab innovator product (Code: CFZ533) in terms of structure and function.

>> Iscalimab biosimilar had been purchased from Abeomics upon their confirmation of its exact sequence identity and resembling all characteristics and functionality of Iscalimab (or CFZ-533).

3. The authors must also disclose if the studies mentioned in the article (lines 61 to 65) were performed with iscalimab biosimilar or with innovator product.

>> The studies cited in our article were performed with iscalimab innovator product.

4. The authors can discuss in depth the impact of this study on the available clinical trial finding in discussion section as additional paragraph. Since, this study justifies why Iscalimab failed in clinical trials, this justification will help the reader.

>> We appreciate that this could be stated more clear. We added a sentence about how our data could explain the limited clinical effect of iscalimab in the revised manuscript (page 12).

“Our data add an alternative explanation why iscalimab showed limited clinical benefit in T cell mediated diseases, where CD40 blockade would be insufficient to offer durable benefit as intervention strategy, as has been shown in clinical trials in transplantation and T1D.”

5. The authors mention funding from Stichting DON but does not disclose whether Iscalimab biosimilar was gifted or bought from the Abeomics.

>> The iscalimab biosimilar used in our study was purchased from Abeomics. We now stated this clearly in the methods section (page 4).

“Iscalimab biosimilar antibody (human IgG anti-CD40, purchased from Abeomics) was used in this study.”

6. The authors mention code CFZ-533 for Iscalimab biosimilar which is also given for Novartis Innovator product. The authors need to rectify this statement.

>> We apologize for this unfortunate glitch and rectified this statement in our revised manuscript.

Reviewer #3

The manuscript examines whether a biosimilar of anti CD40 (iscalimab) modulates effector and naive CD4+ T-cell proliferation in vitro, confirming B-cell inhibition but finding no effect on T-cell responses. The topic is timely and relevant. However, limitations in design, validation, and reporting make it difficult to draw strong mechanistic conclusions. A substantive revision is required before the work can be considered for publication.

Abstract:

Starting with the abstract, which draws a strong clinical conclusion i.e, “iscalimab is unlikely to offer durable benefit as intervention strategy to treat T cell mediated diseases” based solely on in vitro data. This overinterprets the findings. The conclusions should be toned down and framed as hypothesis-generating, with explicit acknowledgment of the limitations of in vitro systems and the heterogeneous outcomes observed in clinical studies.

>> We appreciate that our interpretations of the in vitro data should be toned down into a hypothesis-generating report. We adjusted the abstract (page 1).

“Based on our in vitro data pointing to minimal inhibition of T cells by CD40 blockade, we propose that iscalimab may not suffice to accomplish durable benefit as intervention strategy to treat type 1 diabetes or other T cell mediated diseases.”

Introduction:

The narrative currently relies on clinical trial failures to justify the study and could benefit from a more thorough discussion of alternative explanations (e.g., dosing, pharmacokinetics, patient heterogeneity, disease stage, or tissue targeting). The premise that lack of T-cell inhibition is the main reason for clinical failure is somewhat speculative. Broadening the discussion of CD40 complexity and explicitly acknowledging that clinical inefficacy may result from multiple factors beyond T-cell costimulation would provide valuable context.

>> We agree that alternative factors could also influence the clinical outcomes in the trials besides T cells. We now discuss this in the revised discussion section (page 12).

“The clinical efficacy of iscalimab may be impacted by dosing, pharmacokinetics, patient and disease heterogeneity, disease stage, or affected tissue. Our data add an alternative explanation why iscalimab showed limited clinical benefit in T cell mediated diseases, where CD40 blockade would be insufficient to offer durable benefit as intervention strategy, as has been shown in clinical trials in transplantation and T1D.”

Methods:

Antibody concentration: For PBMC and DC staining, 10 µg/mL iscalimab was used, which is a very high concentration. Please justify this choice. Was a titration performed to determine the minimal concentration required for effective staining?

>> Trough levels of several therapeutic monocloncal antibodies (e.g., rituximab, adalimumab, tocilizumab) are usually around 10µg/ml, whereas intravenous iscalimab reached 848 ug/ml as tough level (PMID 31647605). If anything, the 10µg/mL of iscalimab would be in the low range, but consistent with most therapeutic antibodies. Our previous titration studies on antiCD3 and anti-CD25 in vitro reached complete inhibition of clonal T-cells at 10µg/ml of monoclonal antibodies, for comparison (PMID: 16720206). We state this in the revised manuscript (page 4).

“The concentration (10 µg/ml) was in line with our previous titration studies using anti-CD3 or CD25 to reach complete T-cell inhibition [25], in vitro experiments on B-cell inhibition by iscalimab innovator product by others [19], as well as trough levels in clinical trials assessing rituximab, adalimumab, tocilizumab).”

Viability: Did the authors assess cell viability and exclude dead cells during flow cytometry analysis, as it is essential to avoid artifactual results?

>> Yes indeed, dead cells were excluded from the analyses, which is now stated in the revised manuscript (page 4).

Isotype control: Was a proper isotype control antibody of the same class and at the same concentration used to define positive gates and exclude nonspecific binding? Including such controls is crucial for validating specificity.

>> We agree that controlling for non-specific binding is crucial. To this end, PBMCs and DCs were pre-treated with 50% human serum (corresponding to 4-8mg/ml Ig) to saturate our cultures with all possible Ig, rather than specific isotype control antibody, which should suffice to compete with any non-specific staining using 10µg/ml of anti-CD40.

Secondary antibody: Report the concentration of Mouse anti Human IgG APC secondary antibody used for staining.

>> We apologize for not specifying this; 5µg/ml of Mouse anti Human IgG APC was used for staining with secondary antibody. This has now been specified in the revised manuscript (page 4).

T Cell Models: The study used four long-term cultured, β-cell-specific, autoreactive CD4 T-cell clones. While these are well defined, they represent highly selected, homogeneous effector populations that may not fully reflect the diversity, differentiation states, and costimulatory requirements of T cells in vivo. This selection could influence the detection of CD40 blockade effects and thus the generalisability of the conclusions. Including more physiologically relevant models (e.g., polyclonal or freshly isolated patient T cells) or explicitly acknowledging that this model may underestimate the potential effects of CD40 blockade on other T-cell subsets could further strengthen the study's impact.

>> This is an important point. We agree that testing T cell clones would be a high bar in inhibition experiments. We therefore selected a panel of T cell clones to address a more heterogeneous effector population and also assessed the effect of an iscalimab biosimilar on fully heterogeneous PBL ex vivo to more fully reflect the diversity, differentiation states, and costimulatory requirements of T cells in vivo. No inhibition was observed either PBL or sorted naïve CD4 T-cells (representing a heterogeneous population) in an MLR in the presence of CD40 blockade.

B Cell Inhibition: If possible, include comparative controls (e.g., other CD40 blocking agents) to contextualise potency and specificity.

>> The focus of this report is on iscalimab. B-cells were tested in our studies to confirm that our iscalimab biosimilar showed the expected and desired inhibition of B-cell proliferation as reported before for iscalimab innovator product (CFZ533). We had no intention or reason to compare with other anti-CD40 blocking agents. In our revised manuscript, we added a sentence to leave the possibility that other agents interfering in CD40-CD40L could indeed behave differently than iscalimab, and therefore prove suitable for intervention in T cell mediated diseases (page 12)

Statistics: One-sided paired t-test is used without strong justification, which is generally discouraged unless a clear, a priori directional hypothesis is prespecified. The authors should consider using two-sided tests and apply multiple-comparison adjustments where applicable.

>> Rather than showing a difference (i.e., two-sided), we specifically tested a one-directional affect, namely inhibition. We now stated this a priori justification in our method section describing statistics (page 6).

“Paired student T test (one -sided, expecting reduction) was used to test for inhibition of T cell activation in the presence of iscalimab biosimilar. P value < 0.05 was considered significant.”

Results and Figures

Figures 1, 3, and 4 lack colour legend entries and should be corrected.

>> Agreed. We have now included color legends in the figures themself.

In Figure 1 (monocyte panel), the secondary antibody (mouse-anti-human IgG-APC) shows substantial non-specific binding relative to the no stain control. This should be addres

Attachment Submitted filename: Rebuttal.docx

# Editor report: Decision Letter 1

**DOI:** 10.1371/journal.pone.0351131.r003

**Authors:** Srinivasa Reddy Bonam

24 May 2026

Limited efficacy of a therapeutic anti-CD40 monoclonal antibody to inhibit activated CD4 T cell autoimmunity in vitro

PONE-D-25-64177R1

Dear Dr. Bart O. Roep,

We’re pleased to inform you that your manuscript has been judged scientifically suitable for publication and will be formally accepted for publication once it meets all outstanding technical requirements.

Within one week, you’ll receive an e-mail detailing the required amendments. When these have been addressed, you’ll receive a formal acceptance letter and your manuscript will be scheduled for publication.

An invoice will be generated when your article is formally accepted. Please note, if your institution has a publishing partnership with PLOS and your article meets the relevant criteria, all or part of your publication costs will be covered. Please make sure your user information is up-to-date by logging into Editorial Manager at Editorial Manager® and clicking the ‘Update My Information' link at the top of the page. For questions related to billing, please contact billing support.

If your institution or institutions have a press office, please notify them about your upcoming paper to help maximize its impact. If they’ll be preparing press materials, please inform our press team as soon as possible -- no later than 48 hours after receiving the formal acceptance. Your manuscript will remain under strict press embargo until 2 pm Eastern Time on the date of publication. For more information, please contact onepress@plos.org.

Kind regards,

Srinivasa Reddy Bonam

Academic Editor

PLOS One

# Editor report: Acceptance letter

**DOI:** 10.1371/journal.pone.0351131.r004

**Authors:** Srinivasa Reddy Bonam

PONE-D-25-64177R1

PLOS One

Dear Dr. Roep,

I'm pleased to inform you that your manuscript has been deemed suitable for publication in PLOS One. Congratulations! Your manuscript is now being handed over to our production team.

At this stage, our production department will prepare your paper for publication. This includes ensuring the following:

* All references, tables, and figures are properly cited

* All relevant supporting information is included in the manuscript submission,

* There are no issues that prevent the paper from being properly typeset

You will receive further instructions from the production team, including instructions on how to review your proof when it is ready. Please keep in mind that we are working through a large volume of accepted articles, so please give us a few days to review your paper and let you know the next and final steps.

Lastly, if your institution or institutions have a press office, please let them know about your upcoming paper now to help maximize its impact. If they'll be preparing press materials, please inform our press team within the next 48 hours. Your manuscript will remain under strict press embargo until 2 pm Eastern Time on the date of publication. For more information, please contact onepress@plos.org.

You will receive an invoice from PLOS for your publication fee after your manuscript has reached the completed accept phase. If you receive an email requesting payment before acceptance or for any other service, this may be a phishing scheme. Learn how to identify phishing emails and protect your accounts at https://explore.plos.org/phishing.

If we can help with anything else, please email us at customercare@plos.org.

Thank you for submitting your work to PLOS ONE and supporting open access.

Kind regards,

PLOS ONE Editorial Office Staff

on behalf of

Dr. Srinivasa Reddy Bonam

Academic Editor

PLOS One
